# Comparing the Efficacy of Triamcinolone Acetonide Iontophoresis versus Topical Calcipotriol/Betamethasone Dipropionate in Treating Nail Psoriasis: A Bilateral Controlled Clinical Trial

**DOI:** 10.1155/2018/2637691

**Published:** 2018-11-21

**Authors:** Nasrin Saki, Shahla Hosseinpoor, Alireza Heiran, Ali Mohammadi, Mehdi Zeraatpishe

**Affiliations:** ^1^Molecular Dermatology Research Center, Shiraz University of Medical Sciences, Shiraz, Iran; ^2^Dermatology Department, Shiraz University of Medical Sciences, Shiraz, Iran; ^3^Student Research Committee, Shiraz University of Medical Sciences, Shiraz, Iran

## Abstract

**Background and Objective:**

Psoriasis is a common chronic inflammatory skin disorder affecting any age and gender. The clinical presentation of the nail disease depends on the location of the pathology: nail bed or nail matrix. We aimed to compare the therapeutic effects of triamcinolone acetonide iontophoresis (TI) and topical calcipotriol/betamethasone dipropionate in the nail bed and nail matrix involvements of psoriasis using Nail Psoriasis Severity Index (NAPSI).

**Materials and Methods:**

In the present bilateral comparison clinical trial, sixteen patients with clinical diagnosis of nail psoriasis were enrolled and randomized to receive six monthly TI treatment sessions either on their right or on the left hand target nails and daily application of topical calcipotriol/betamethasone dipropionate for six months on their other hand. Clinical efficacy was evaluated according to target nails NAPSI before and after the treatment. Wilcoxon sign-rank test and repeated measures ANOVA were used to compare the efficacy of the treatments.

**Results:**

The results did not show any difference between the therapeutic effects of TI and topical calcipotriol/betamethasone dipropionate regarding the nail bed score (*P* value =  .356), matrix score (*P* value =  .137), and total NAPSI (*P*-value =  .098).

**Conclusion:**

Monthly TI has an equal efficacy compared to daily topical calcipotriol/betamethasone dipropionate. It can be used as a safe, easy, and compliant treatment for nail psoriasis. This study is registered under IRCT2017050233778N1.

## 1. Introduction

Psoriasis is a common chronic inflammatory skin disorder affecting any age or gender [1]. It may involve the extensor surfaces, scalp, joints, creases, or nails. Nail disease can occur even in patients without skin involvements [2]. Although nails account for a small proportion of the body surface area, psoriasis on such visible parts of the body, such as the face and hands, has a major negative impact on physical, psychological, and social aspects of the patient's quality of life [3]. As a result, treatment of nail psoriasis, whether skin is involved or not, is a topic of concern.

Regarding the location of the nail pathology, the clinical presentation is different; pitting, leukonychia, red spots in the lunula, nail plate crumbling, beaus lines, and trachyonychia are signs of nail matrix involvement and onycholysis, oil drop discoloration or salmon patch, subungual hyperkeratosis, and splinter hemorrhage indicate nail bed disease. Matrix involvement is more recalcitrant to the therapeutic options compared to nail bed disease [4]. Due to the significant impact of nail involvement diagnosis on the outlook and severity of psoriasis, dermatologists put Nail Psoriasis Severity Index (NAPSI) in their toolbox to score the disease severity and evaluate the treatment response. NAPSI is a numeric, reproducible, objective, and simple tool for evaluation of the severity of nail bed and nail matrix psoriasis based on the area of involvement in the nail unit [5–7].

Treatment options for nail psoriasis depend on various factors including clinical presentations and patient-related factors [8] and are aimed to inhibit epidermal proliferation, inflammation, or both [4]. They include topical and systemic agents, biologic drugs, phototherapy, intralesional corticosteroid injection, or pulse dye laser [9, 10]. Iontophoresis is a delivery system which enhances the absorption and movement of different metal ions or drugs across biological tissues, such as the skin, muscles, tendons, and joints via a low power electrical current [11].

Triamcinolone acetonide (TI) is a synthetic corticosteroid used by several routes; topical, intramuscular, or intravenous injections, for different therapeutic aims in dermatology [12].

Topical calcipotriol/betamethasone dipropionate is a yellow-to-white substance including 50* μg/g* calcipotriol and 500* μg/g* betamethasone dipropionate. Calcipotriol is a vitamin D derivative and acts similar to vitamin D, making a reversible temperature-dependent equilibrium between calcipotriol and precalcipotriol. This drug is recommended for once daily application in treating plaque-type psoriasis [13].

With regard to anatomical structure of the nail, achieving sufficient concentrations of topical antipsoriatic drugs in the nail plate, nail bed, or nail matrix is an issue; therefore, innovative methods providing adequate penetration of the agents are desirable. In this study, we aimed to compare the therapeutic efficacy between TI iontophoresis and topical calcipotriol/betamethasone dipropionate regarding nail bed score, matrix score, and total NAPSI.

## 2. Materials and Methods

### 2.1. Participants

This bilateral comparison clinical trial was conducted on sixteen patients with bilateral nail psoriasis referred to the Faghihi Hospital Dermatology Clinic, Shiraz, Iran, affiliated to University of Medical Science from March 2015 to March 2016. Patients with mild-to-moderate nail psoriasis were enrolled in the study after dermatologist clinical diagnosis confirmation and tissue biopsy. Patients who were on systemic medications did not stop their drugs considering the fact that the bilateral design resolves the demographic and baseline matching problems.

Exclusion criteria were having pacemakers, pregnancy, metal orthopedic implant, intrauterine device (IUD), and cardiac arrhythmia, hypersensitivity to any assigned medication and patients' dissatisfaction.

This study was approved by the local Ethics Committee of Shiraz University of Medical Sciences (code: IR.SUMS.MED.REC.1396.37) and registered by the Iranian Registry of Clinical Trials (code: IRCT2017050233778N1). All the patients signed the written informed consent form prior to initiation of the trial.

### 2.2. Materials and Procedures

Hand randomization for each patient was carried out through flipping a coin to receive six monthly scheduled triamcinolone acetonide (Sina Darou, Iran) iontophoresis treatment sessions either on the right or on the left hand target nails and daily application of calcipotriol/betamethasone dipropionate ointment (Leo Pharma, Ltd) for six months on the other hand.

The investigator dermatologist conducted the iontophoresis procedure in Faghihi Hospital Dermatology Clinic. To perform Iontophoresis (Irantronics Co., Iran), a low power electrical current was exploited to deliver triamcinolone into the skin. Fifty* ml* distilled water combined with 5* ml* triamcinolone (40* mg/ml*) as a homogenous mixture (final concentration of 4* mg/ml*) was prepared and put in a shallow plastic container in which all fingernails of a hand were dipped. The current of 4* mA* (pulse duration of 0.2 second) was passed through the solution for 20 minutes.

### 2.3. Clinical Assessments

At baseline a questionnaire was filled for each patient including demographic data, degree of skin involvement, and systemic medication consumption.

NAPSI of both hands' fingers was calculated at baseline and six monthly follow-ups by a well-trained dermatologist in line with six monthly triamcinolone acetonide iontophoresis sessions and daily application of calcipotriol/betamethasone dipropionate ointment for six months. To calculate NAPSI of a hand, nail plates were divided into four quadrants by imaginary longitudinal and horizontal lines. Each nail plate/bed was scored through 0-4; 0 if a nail plate/bed was intact and 4 if nail plate/bed involvement was present in all 4 quadrants; thus each nail has a matrix score (0-4) and a nail bed score (0-4), and the total nail score is 0-8. NAPSI of a hand was the sum of the total score of all fingernails (0-40).

### 2.4. Statistical Analysis

We used SPSS statistical package (IBM Corp. Released 2012. IBM SPSS Statistics for Windows, Version 21.0. Armonk, NY: IBM Corp.). The Wilcoxon sign-rank test was used to compare the efficacy of TI and topical calcipotriol/betamethasone dipropionate. Repeated measures ANOVA were applied to analyze the pattern of expected reduction in each treatments, separately.* P value* ≤ 0.05 was considered as statistically significantly different.

## 3. Results

**Figure 1:**
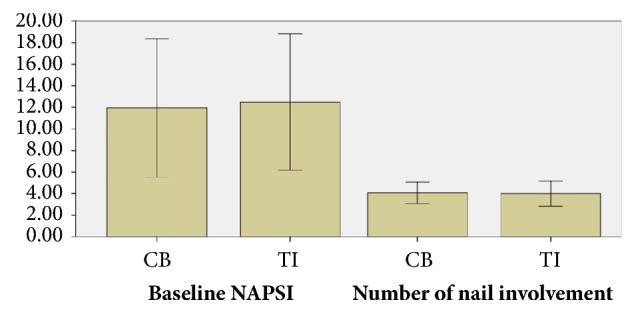
Baseline characteristics; TI: triamcinolone acetonide iontophoresis; CB: calcipotriol/betamethasone dipropionate.

**Figure 2:**
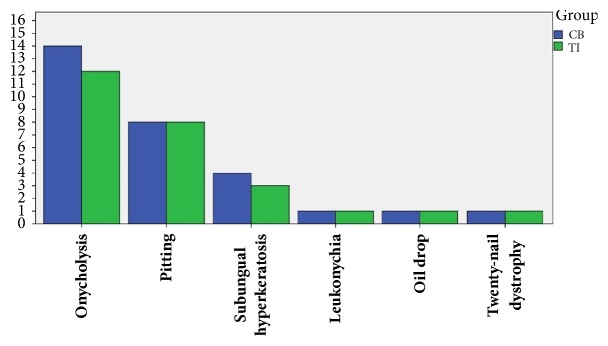
Patient's nail findings; TI: triamcinolone acetonide iontophoresis; CB: calcipotriol/betamethasone dipropionate.

The baseline features and nail findings are depicted in Figures 1 and 2. Sixteen patients completed the study: eleven females (68.8%) and five males (31.2%). No patient discontinued the study. The mean age was 34.31 ± 17.3 (ranged between 6 and 70 years of age). Three of them (18.8%) had skin involvement and two patients (12.5%) had positive history of systemic medication consumption.

Four patients (25%) showed only bed involvement, two patients (12.5%) had only matrix involvement, and ten patients had both bed and matrix involvements. The minimum and the maximum number of nail involvements were four and ten, and the mean number of nail involvement in hands treated with TI and topical calcipotriol/betamethasone dipropionate were 4 ± 1.15 and 4.07 ± 1.

At baseline, there was not difference between TI or calcipotriol/betamethasone dipropionate groups, regarding nail bed score (TI: 5.63 ± 3.59 versus calcipotriol/betamethasone dipropionate: 5.5 ± 3.5;* P* value =  .836), matrix score (TI: 6.88 ± 8.16 versus calcipotriol/betamethasone dipropionate: 6.44 ± 8.49;* P* value =  .672), and NAPSI (TI: 12.5 ± 6.31 versus calcipotriol/betamethasone dipropionate: 11.94 ± 6.42;* P* value =  .472).

Initial NAPSI reduction by TI after third and fourth follow-up was recorded for 7 (43.75%) and 13 (81.25%) patients, while it was achieved by topical calcipotriol/betamethasone dipropionate in 3 (18.75%) and 10 (62.5%) patients. Failure to both treatments (constant NAPSI) was observed in a patient with twenty-nail dystrophy. Bilateral complete response (NAPSI of zero) was observed in three patients.

**Figure 3:**
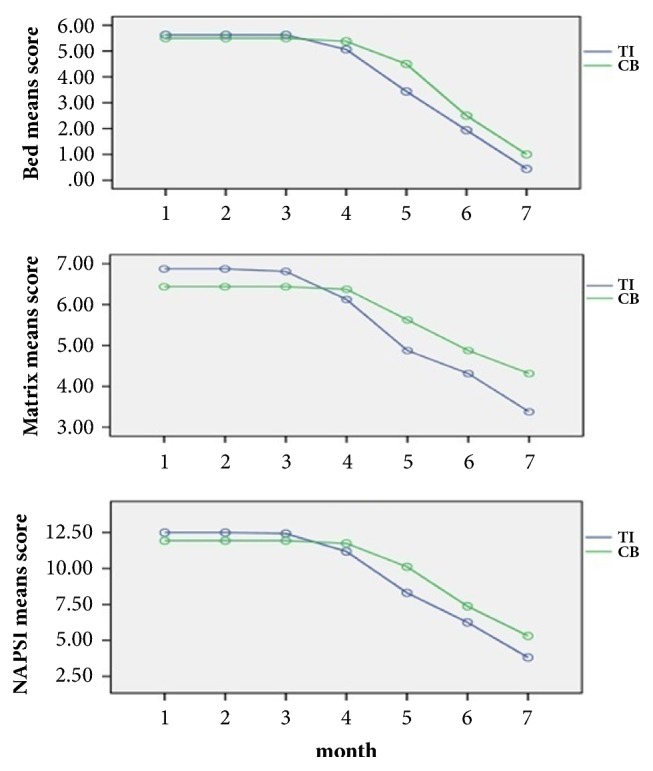
Mean nail bed, matrix, and NAPSI score reduction over the study; TI: triamcinolone acetonide iontophoresis; CB: calcipotriol/betamethasone dipropionate. Numbers 1 and 7 are indicator of baseline and 6 months.

When comparing TI and calcipotriol/betamethasone dipropionate for the overall decrease (∆) in severity score, no difference between the therapeutic effect of TI and topical calcipotriol/betamethasone dipropionate was observed, regarding the nail bed score (TI (∆): −5.19 ± 3.25 versus calcipotriol/betamethasone dipropionate (∆): −4.5 ± 3.46,* P* value =  .356), matrix score (TI (∆): −3.5 ± 4.83 versus calcipotriol/betamethasone dipropionate (∆): −2.13 ± 4.70,* P* value =  .137), and NAPSI (TI (∆): −8.69 ± 4.05 versus calcipotriol/betamethasone dipropionate (∆): −6.63 ± 4.26,* P* value =  .098) (Figure 3, Table 1).

The mean ± SD of nail bed score, matrix score, and NAPSI at the baseline and six monthly follow-up visits for both treatments were shown at Table 1 and Figure 3.

Considering the interventions separately, in the hands treated with TI a diminution pattern was observed for nail bed score (*P* value <  .001), matrix score (*P* value =  .011), and NAPSI (*P* value <  .001), and in the hands treated with topical calcipotriol/betamethasone dipropionate the same trend was observed for nail bed score (*P* value <  .001) and NAPSI (*P*-value <  .001) but not for matrix score (*P* value =  .104).

**Figure 4:**
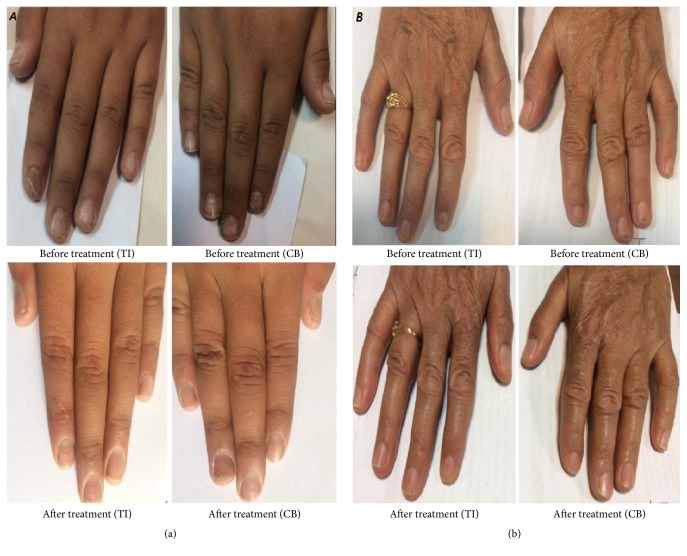
(a) A patient with nail matrix involvement (pitting); (b) patient with nail bed involvement (onycholysis); TI: triamcinolone iontophoresis; CB: calcipotriol/betamethasone dipropionate.

Patients' satisfaction was evaluated by a visual analog scale (VAS) and no difference between the two therapies was observed (*P* value =  .42).  Figure 4 depicts the improvement of matrix lesion (pitting) and nail bed lesion (onycholysis) over the study.

## 4. Discussion and Conclusion

The puzzling treatment of nail psoriasis still remains unanswered, since topical treatments minimally influence nail involvements and systemic therapies are accompanied with several side effects such as hepatotoxicity, hypertension, renal dysfunction, immunosuppression, and severe infections, and hence they are not suitable for those with mild nail psoriasis without severe skin involvements [10, 14–16].

In a recently published review study on treatment strategies, all biologic agents including antitumor necrosis factor-a, anti-interleukin (IL) 17, and anti-IL-12/23 antibodies were introduced as the highly effective available therapies, followed by systemic therapies comprising methotrexate, cyclosporine, acitretin, and apremilast, as well as intralesional corticosteroids. In mild cases, topical treatments, including corticosteroids, calcipotriol, tacrolimus, and tazarotene could be applied. Finally, Pasch discussed the present heterogeneity of outcome measures and scarcity in trials and addressed the demand on more studies [2].

The key factor in treating nail psoriasis is to accumulate the therapeutic concentration of pharmacological agent into the site of psoriatic inflammation, the nail bed, or the nail matrix. Due to the low permeability of most drugs across the nail plate and challenging structural issues in the nail diseases, many efforts have been made to invent creative methods to set up the efficient concentration of so-called safe topical therapies with marginal side effects.

The iontophoresis, a physical method to enhance nail penetration, perhaps decreases the treatment time and enhances the efficacy by increasing drug molecules transport across and into the nail plate and the consequent higher concentration of drugs. Additionally, this technique might be a more rapid-more concentrated alternative delivery system to oral route in drugs which are poorly soluble in water through lipophilicity augmentation of many molecules, as it is applied after ablative laser procedures or variety of other cosmetic procedures [17].

Studies investigating iontophoresis efficacy on nail diseases are scarce. In a study published in 2012, Van Le and Howard [18] investigated dexamethasone iontophoresis efficacy on twenty-seven patients with nail psoriasis. After all, twenty-two (81%) patients showed clinical improvement and NAPSI reduction with mean improvement score and mean duration for initial response (month) of eight and four, respectively. Terbinafine, conventionally orally taken, is regarded as a main therapy for onychomycosis, but side effects and poor solubility in water make iontophoresis a rational alternative modality to deliver terbinafine to the nails. Indeed consecutive studies showed promising findings in treating onychomycosis [19, 20]. However, more studies on different drugs are required to get conclusive results regarding the clinical efficacy of iontophoresis in treating nail diseases and this field is an untraveled road.

To the best of our knowledge, the present research is first study comparing iontophoresis with another modality, and first triamcinolone acetonide iontophoresis evaluation as well. Additionally, as an advantage each patient served as her/his own control to reduce confounding factors and personal differences. The present study showed that both treatments improved the overall and Patients did not develop any side effects.

TI was effective in treating nail bed and matrix lesions during the 6-month course of treatment with superior impact on nail bed lesions and more rapid onset of improvement compared to topical calcipotriol/betamethasone dipropionate. Studies on intralesional injection of triamcinolone acetonide [9, 21–26] showed that this modality is particularly effective for improving nail matrix lesions and moderately effective for nail bed lesions [2]. But intralesional injection is a painful procedure and accompanied with numerous reported adverse effects such as subungual hematomas, short-term paresthesia, loss of nail plate, atrophy at the injection sites, epidermal inclusion cysts, tattooing with minute rubber particles, rupture of extensor tendon, and blood splash-back on the instrument and the physician [2, 9, 21, 25–28]; hence, this procedure cannot be a preferred choice of treatment. In addition, our findings were obtained from more severe cases, higher mean baseline NAPSI, compared with milder nail psoriasis patients enrolled in most of the aforementioned studies on intralesional steroid injections.

Combination therapy with corticosteroids, particularly the combination of topical betamethasone with calcipotriol, has been investigated in several studies [16, 18, 29, 30] and currently is used as a first line treatment for nail psoriasis, especially bed involvements. In a recent research performed by Rigopoulos et al. [29], twenty-five psoriatic patients with nail and mild skin involvements were instructed to apply daily combined calcipotriol-betamethasone ointment for 12-weeks on affected nails. Outcome measures were assessed by NAPSI at baseline and 4th, 8th, and 12th weeks. Overall improvement was 72% reduction in NAPSI (∆: −4.2). Significant improvement for hyperkeratosis and onycholysis, moderate improvement for oil drops, and slight improvement for pitting were observed. However, such results were obtained from mild nail psoriasis cases since baseline mean NAPSI was 5.8. This result was almost in line with our study that topical calcipotriol/betamethasone dipropionate had a significant effect on nail bed lesions, but such trait was not found for the matrix lesions. The overall reduction obtained with topical calcipotriol/betamethasone dipropionate was greater with regard to the more treatment sessions in our study.

Patients did not develop any side effects in our study. Besides the bilateral comparison design and side effect free treatments, one of the TI advantages was monthly sessions; hence patients' time was saved, the procedure was cheaper, and they had a better compliance.

We found that monthly TI has equal efficacy compared to daily topical calcipotriol/betamethasone dipropionate in improving the nail bed score, matrix score, and NAPSI. It can be used as a safe and easy treatment for nail psoriasis. However, knowledge on iontophoresis in treating nail psoriasis is scarce and more studies in this field are needed. We recommend conducting studies focusing on the other aspects of nail psoriasis iontophoresis including use of other drugs to find an optimum iontophoresis protocol for nail psoriasis [31, 32].

## Figures and Tables

**Table 1:** Mean severity score reduction during the course of study.

Treatment	Anatomical site	Mean severity score ± SD							*P*-value
		Measurement number							
		0	1	2	3	4	5	6	
	Nail bed score	5.63 ± 3.59	5.63 ± 3.59	5.63 ± 3.59	5.06 ± 3.49	3.44 ± 2.68	1.94 ± 2.02	0.44 ± .89	< .001
TI	Matrix score	6.88 ± 8.16	6.88 ± 8.16	6.81 ± 8.1	6.13 ± 7.77	4.88 ± 7.06	4.31 ± 6.77	3.38 ± 5.91	.011
	NAPSI	12.5 ± 6.31	12.5 ± 6.31	12.44 ± 6.3	11.19 ± 6.07	8.31 ± 5.9	6.25 ± 6.44	3.81 ± 5.86	< .001

	Nail bed score	5.5 ± 3.5	5.5 ± 3.5	5.5 ± 3.5	5.38 ± 3.42	4.5 ± 3.37	2.5 ± 2.48	1 ± 1.71	< .001
CB	Matrix score	6.44 ± 8.49	6.44 ± 8.49	6.44 ± 8.49	6.38 ± 8.38	5.63 ± 7.56	4.88 ± 7.22	4.31 ± 6.81	.104
	NAPSI	11.94 ± 6.42	11.94 ± 6.42	11.94 ± 6.42	11.75 ± 6.42	10.13 ± 5.84	7.38 ± 6.65	5.31 ± 6.68	< .001

TI: triamcinolone acetonide iontophoresis, CB: calcipotriol/betamethasone dipropionate, and SD: standard deviation.

## Data Availability

The data used to support the findings of this study are included within the article.
